# Lineage-specific expansion of proteins exported to erythrocytes in malaria parasites

**DOI:** 10.1186/gb-2006-7-2-r12

**Published:** 2006-02-20

**Authors:** Tobias J Sargeant, Matthias Marti, Elisabet Caler, Jane M Carlton, Ken Simpson, Terence P Speed, Alan F Cowman

**Affiliations:** 1 The Walter and Eliza Hall Institute of Medical Research, Melbourne, Victoria 3050, Australia; 2 Department of Medical Biology, The University of Melbourne, Parkville, Victoria 3010, Australia; 3 The Institute for Genomic Research (TIGR), Rockville, Maryland 20850, USA

## Abstract

**Background:**

The apicomplexan parasite *Plasmodium falciparum *causes the most severe form of malaria in humans. After invasion into erythrocytes, asexual parasite stages drastically alter their host cell and export remodeling and virulence proteins. Previously, we have reported identification and functional analysis of a short motif necessary for export of proteins out of the parasite and into the red blood cell.

**Results:**

We have developed software for the prediction of exported proteins in the genus *Plasmodium*, and identified exported proteins conserved between malaria parasites infecting rodents and the two major causes of human malaria, *P. falciparum *and *P. vivax*. This conserved 'exportome' is confined to a few subtelomeric chromosomal regions in *P. falciparum *and the synteny of these and surrounding regions is conserved in *P. vivax*. We have identified a novel gene family PHIST (for *Plasmodium *helical interspersed subtelomeric family) that shares a unique domain with 72 paralogs in *P. falciparum *and 39 in *P. vivax*; however, there is only one member in each of the three species studied from the *P. berghei *lineage.

**Conclusion:**

These data suggest radiation of genes encoding remodeling and virulence factors from a small number of loci in a common *Plasmodium *ancestor, and imply a closer phylogenetic relationship between the *P. vivax *and *P. falciparum *lineages than previously believed. The presence of a conserved 'exportome' in the genus *Plasmodium *has important implications for our understanding of both common mechanisms and species-specific differences in host-parasite interactions, and may be crucial in developing novel antimalarial drugs to this infectious disease.

## Abstract 2

A new software was used to predict exported proteins that are conserved between malaria parasites infecting rodents and those infecting humans, revealing a lineage-specific expansion of exported proteins.

## Background

*Plasmodium falciparum *is the causative agent of the most virulent form of malaria in humans, causing major mortality and morbidity in populations where this disease is endemic. Several other species of *Plasmodium *infect humans, including *P. vivax*, *P. malariae *and *P. ovale*. Species of the genus *Plasmodium *are obligate intracellular parasites, switching between an arthropod vector and their respective vertebrate host, where they undergo cycles of asexual reproduction in erythrocytes. The infected erythrocytes are subject to an extensive remodeling process induced by the parasite, which facilitates surface exposition of various ligands for host cell receptors, nutrient import into the parasite and asexual reproduction within the host cell. Host cell remodeling includes the development of electron dense protrusions on the infected red blood cell surface called knobs. Knob-associated histidine-rich protein (KAHRP) is a structural knob component that anchors the major virulence factor *Plasmodium falciparum *erythrocyte surface protein 1 (PfEMP1) on the knob surface [1]. PfEMP1 is encoded by the epigenetically regulated *var *multigene family, and is implicated in cytoadherence of infected red blood cells to various host cells; a causative factor in the severe pathology of the disease [2-4]. Recently, a second gene family encoding surface antigens has been described, the repetitive interspersed family (*Rif*) and it is believed that Rifins are also subject to antigenic variation [5].

Once inside the infected erythrocyte the parasite resides in a parasitophorous vacuole, which acts as a biochemical barrier between parasite and host through which parasite proteins must be translocated to reach the parasite-infected erythrocyte cytosol and the host cell membrane. It has recently been shown that transport of parasite proteins via the parasitophorous vacuole and into the host cell depends on a short amino-terminal sequence, R/KxLxE/Q [6,7], which we have termed PEXEL (for Plasmodium export element).

This sequence is functionally conserved across the genus *Plasmodium*, indicating the presence of a conserved export mechanism across the parasitophorous vacuole membrane in malaria parasites. The PEXEL sequence has allowed the prediction of proteins exported into the host erythrocyte, which are likely to be important to both erythrocyte remodeling and virulence. The availability of genome sequences from many different species of the genus *Plasmodium *now provides an opportunity for the genus-wide discovery of exported proteins and for the identification of specific protein domains representing conserved functions in these different organisms.

Here we have developed and applied a method to systematically identify exported proteins in the genus Plasmodium and to allow characterisation of the 'exportome' in the three most characterised *Plasmodium *lineages: *P. falciparum/P. reichenowi *(the '*P. falciparum *lineage') and *P. vivax/P. knowlesi *(the '*P. vivax *lineage'), encompassing parasites that infect primates, and *P. berghei*/*P. yoelii*/*P. chabaudi *(the '*P. berghei *lineage') with parasites infecting rodents. We identified a core set of exported proteins conserved across the genus *Plasmodium *that are predicted to play key functions in the host cell remodeling process. Additionally, we describe a set of novel gene families encoding exported proteins likely to be important in the differential properties of the genus *Plasmodium *in their respective host cells.

## Results

### ExportPred: algorithmic prediction of the *P. falciparum *exportome

Previous strategies [6,7] to determine the complement of *Plasmodium *proteins exported to the parasite-infected erythrocyte by predicting the presence of a signal sequence and a functional PEXEL element have seriously underestimated the full complement of exported proteins. A significant number of secreted *P. falciparum *proteins have a hydrophilic spacer of up to 50 amino acids preceding the hydrophobic signal sequence, referred to as a recessed signal sequence. Functional *P. falciparum *signal sequences, especially those that are recessed, can be mispredicted by SignalP [8], resulting in a large deficit in the number of exported proteins [7]. Other methods to determine the full exportome have limitations and do not provide a statistic that can be used to gauge the likelihood of export. To identify the exportome of *P. falciparum*, and other species of the genus *Plasmodium*, we constructed an algorithm for export prediction. This algorithm, named ExportPred, uses a generalised hidden Markov model (GHMM) [9] to model simultaneously the signal sequence and PEXEL motif features required for protein export.

**Figure 1:**
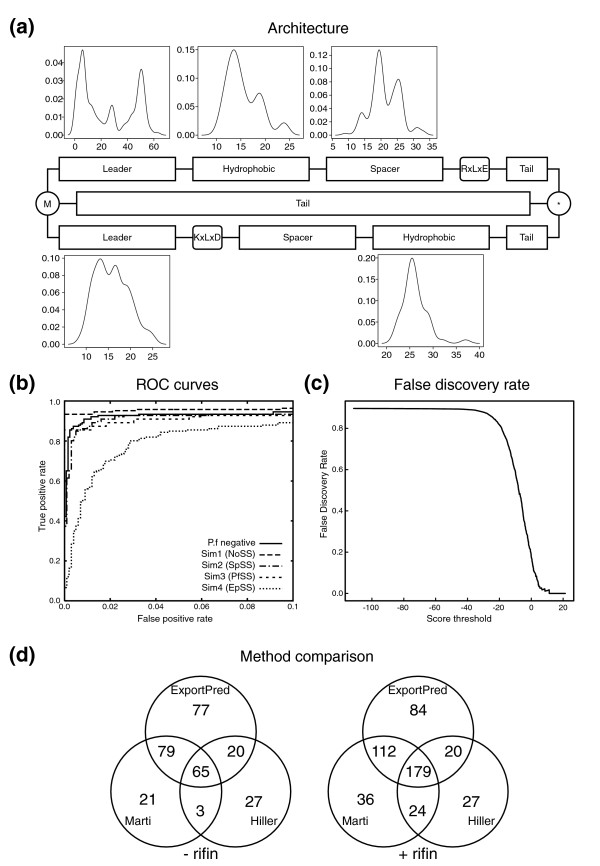
ExportPred: Architecture and performance. **(a) **The architecture of the ExportPred GHMM. The GHMM progresses from left to right, beginning with an amino-terminal methionine and terminating at a stop codon. Length probability densities are shown for non-geometric states. Tail states and the KLD spacer state are modelled by geometric distributions. **(b) **ROC curves for the ExportPred model comparing the training against the five described negative sets. **(c) **False discovery rate as a function of score threshold, calculated using the training set and the *P.f *negative set, and assuming 10% of the *P. falciparum *proteome is exported. **(d) **Comparison of predictions made by ExportPred using the default threshold of 4.3 with those published in [6,7]. The -rifin set is exclusive of any sequence annotated as rifin or stevor, whereas the +rifin set includes these sequences.

**Figure 2:**
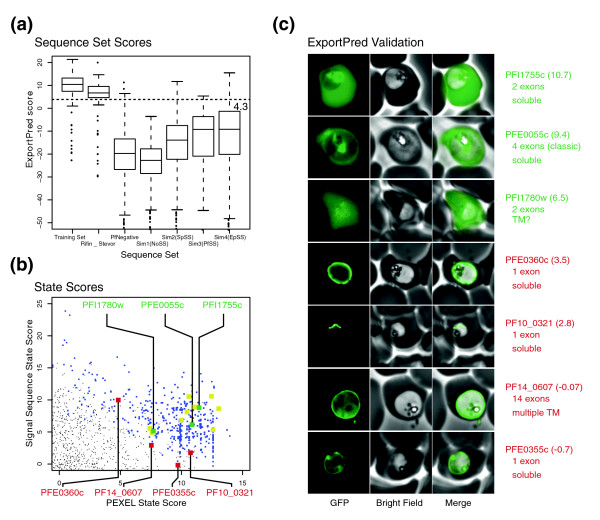
ExportPred: Training sets and validation. **(a) **Boxplots of scores of two positive sequence sets and five negative sequence sets. The chosen score threshold of 4.3 is marked. Both positive sets are well separated from all negative sets. Poorly scoring outliers in the postive sets can largely be ascribed to incorrect gene models and *Rif *and *Stevor *pseudogenes. **(b) **Two-dimensional plot of *P. falciparum *proteins decomposed by scores of the ExportPred states for the PEXEL motif and for the signal sequence. Small black dots indicate proteins with full model scores <4.3 and blue dots with scores ≥ 4.3. The three positive and four negative GFP fusions described are marked with green and red dots, respectively, and the nine yellow dots are, from left to right, RESA, HRPIII, KAHRP, PFA0475w (Rifin), R45, MESA, PfEMP3, PFC0025c (Stevor), and GBP130. **(c) **Experimental verification of a number of ExportPred predictions above (green) and below (red) the chosen threshold. GFP fusions to three positive predictions (PFI1780w, PFE0055c, PFI1755c) are exported successfully into the red blood cell cytosol. Fusion proteins to three negative predictions (PFE0360c, PF14_0607, PFE0355w) accumulate in the parasitophorous vacuole, indicating a functional signal sequence but no functional export motif. One GFP fusion (PF10_0321) appears to be targeted to the mitochondrion. ExportPred scores are indicated in parentheses.

Figures 1b and 2a demonstrate that ExportPred is able to distinguish exported proteins from those that are not exported. To test both the effect of our simplified signal sequence model and PEXEL motif, we substituted the signal sequence portion of the ExportPred GHMM with the HMM used in SignalP and the motif portion with the weight matrix [7]. Combinations of these substitutions gave rise to three new versions of ExportPred. Table 1 lists the discriminatory power of these various model configurations and positive and negative sets as measured by area under the respective Receiver Operating Characteristic (ROC) curve. Variants of ExportPred tend to perform less well than the standard ExportPred model, even after augmenting the SignalP model to allow for recessed signal sequences. The inclusion of the alternative weight matrix does not improve discrimination in any of the cases examined and, in fact, appears to result in a decrease in accuracy in many cases.

### Validation of ExportPred

To provide *in vivo *support for the ExportPred predictions, we generated a series of green fluorescent protein (GFP) fusions to unknown proteins conserved in *Plasmodium *that were ranked highly in the ExportPred output. Proteins were chosen to test various properties of exported proteins, including number of exons in the encoding gene, motif composition and presence of multiple transmembrane domains. As in our initial study [6], we fused the native amino terminus including the predicted PEXEL plus 11 amino acids downstream of it to GFP, since it has been shown that a spacer between the motif and a reporter is needed for correct export [10]. Figure 2b shows the seven GFP chimeras created in this study in the context of nine known exported proteins and the positive and negative ExportPred predictions from the *P. falciparum *proteome. Each protein sequence is represented by a point in two-dimensional space determined by the contributions to the ExportPred score of the predicted signal sequence and the PEXEL.

**Figure 3:**
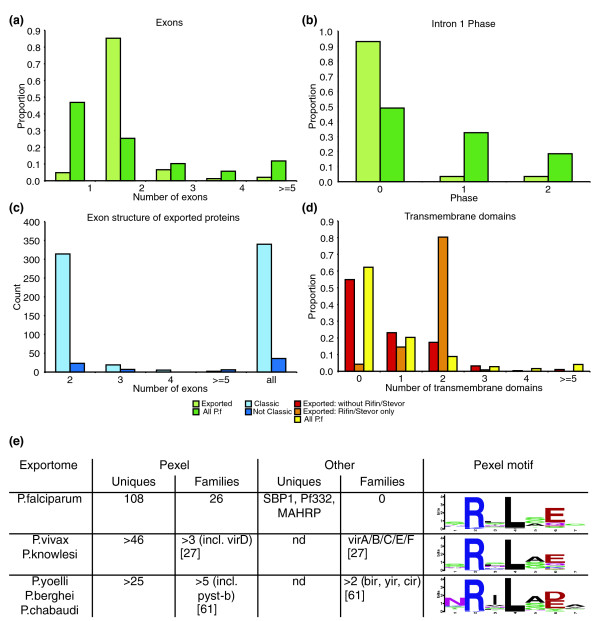
*Plasmodium *'exportome' statistics. **(a) **Distribution of exon counts in genes with PEXEL export signatures compared with all *P. falciparum *genes, demonstrating a clear trend towards two exon genes in the *P. falciparum *exportome. **(b) **First intron phase for PEXEL exported genes compared with all *P. falciparum *genes, showing an extremely strong trend towards a phase 0 first intron amongst genes with export signatures. **(c) **Counts of classic (intron between signal sequence and PEXEL) and non-classic genes in the *P. falciparum *exportome, stratified by exon count. **(d) **Distributions of the number of predicted transmembrane domains for exported *P. falciparum *proteins, Rifins and Stevors, and the *P. falciparum *proteome as a whole. Rifins and Stevors are, in general, predicted to have two transmembrane domains, and members of the remaining complement of the *P. falciparum *exportome are slightly less likely to be soluble than *P. falciparum *proteins in general, and are also less likely to be multi-membrane spanning. **(e) **Comparison of the *P. falciparum *exportome with hybrid exportomes of the *P. vivax *and *P. berghei *lineages. Numbers of PEXEL exported uniques and families are shown, as well as any previously described families and uniques not apparently exported by PEXEL mediated mechanisms. Web logos constructed from instances of the motif in the three exportomes are also shown. References to gene families from species other than *P. falciparum *are indicated in brackets.

PF14_0607 is predicted to be a multispanning membrane protein encoded by a 14-exon gene, both features suggesting it was unlikely that the protein was exported (Figure 3). The protein has a negative ExportPred score because of a suboptimal signal sequence prediction and an unusual amino acid (phenylalanine) in position 4 of the motif. The fusion protein accumulated in the parasitophorous vacuole rather than being exported, demonstrating that the amino terminus could not mediate export (Figure 2c). Next, we tested two proteins encoded by single exon genes located in tandem in the central region of chromosome 5. *PFE0355c *encodes the putative serine protease PfSubtilisin 3, the least characterised member of the *Plasmodium *subtilisin protease family. Both PfSubtilisin 1 and 2 have been described as merozoite proteins and, at least for PfSubtilisin 1, there is accumulating evidence for localisation of the mature protein in the dense granules [11]. PFE0355c has an unusually long spacer between the signal sequence and the predicted PEXEL motif, which resulted in a negative ExportPred prediction; in agreement with this the fusion protein accumulated in the parasitophorous vacuole (Figure 2c). The single exon gene *PFE0360c *encodes a protein of unknown function and had a positive ExportPred score (3.49 in PFE0360c) for all *Plasmodium *species where the ortholog was found. However, the motif has an unusual amino acid, glutamic acid, in position 4 and the fusion protein accumulates in the parasitophorous vacuole rather than being exported (Figure 2c). *PF10_0321 *is also a single exon gene encoding a protein of unknown function. Although the export motif was close to the consensus, the short hydrophobic amino terminus was not predicted to be a signal sequence. The fusion protein localised to the mitochondrion (Figure 2c) and, indeed, the amino terminus is predicted to be a mitochondrial transit peptide (91% predicted with PlasMit [12]). We also tested a number of positively predicted export motifs. PFE0055c is a four-exon gene encoding a putative type I DnaJ protein (that is, containing all three DnaJ domains, see below). It had a high PEXEL score and the fusion protein was exported into the parasite-infected erythrocyte. PFI1780w has a two-exon structure and encodes a protein of unknown function, which may have multiple transmembrane domains. Importantly, it contains one of the few predicted PEXEL motifs with a lysine rather than an arginine in position 1 (except for the PfEMP1-type motif, where it is the rule). The fusion protein was clearly exported and distributed evenly in the host cell cytoplasm. Finally, we made a GFP fusion to PFI1755c, one of the most highly expressed asexual stage proteins [13,14]. It is encoded by a two-exon gene located adjacent to PFI1780w on chromosome 9; the encoded protein has a high ExportPred score and, as expected, the GFP chimera was efficiently exported to the infected-erythrocyte cytoplasm (Figure 2c). As expected, the presence of a signal sequence in the absence of a predicted PEXEL resulted in accumulation of the reporter protein in the parasitophorous vacuole. Taken together, these data show that ExportPred can accurately predict functional PEXEL motifs.

### The *P. falciparum *exportome

Using as input all *P. falciparum *annotations and automatic gene prediction, ExportPred predicted 797 sequences as being exported, many of which represent overlapping gene predictions and annotations. To address the issue of misannotation of genes, we selected the highest scoring model in each overlapping group and some were inspected manually. After curation, 59 predictions with a PfEMP1-type motif (the whole PfEMP1 set encoded in 3D7 except var2CSA) and 396 predictions with a generic motif with score ≥ 4.3 remained (see Additional data file 2 for a detailed list). The structures of the 396 predicted genes show a strong tendency towards two exons (Figure 3a) and, in 93% of cases, the first intron occurs in phase 0 (Figure 3b). Inspecting the GHMM state in which the first intron occurs indicates that in 90% of cases the first intron occurs in the spacer between the signal sequence and the PEXEL motif, or, less commonly, late in the hydrophobic stretch (>75% of signal sequence in the first exon), confirming that the majority of PEXEL containing genes have a similar structure, with the signal sequence in the first exon divided from the export motif by an intron in phase 0 (Figure 3c). Many proteins in the exported proteome of *P. falciparum *have one or two predicted transmembrane domains (Figure 3d). Only four sequences were predicted to possess more than three transmembrane regions.

To cluster the 396 predicted genes into putative families, we performed an all by all comparison to generate pairs of reciprocal BLAST hits (see Material and methods). This approach yielded 26 families shown in Table 2: 16 families encode hypothetical proteins containing novel domains, while others have been previously described, such as the Rifin [5,15] and Stevor[16] families, a family of Maurer's clefts localised proteins termed PfMC-2TM (Maurer's clefts two transmembrane protein family [17]) and a family of putative protein kinases (denoted FIKK kinases) [18,19]. Two of the novel families encode DnaJ domains and another two a/b hydrolase domains. In total, at least 287 of 396 exported proteins are members of families - approximately 75% of the exportome.

### A core set of proteins are conserved in the *Plasmodium *exportome

One of the major goals of this study was to determine whether a subset of exported proteins conserved across the genus *Plasmodium *exists. Since PEXEL-mediated protein export appears to be functionally conserved across Plasmodia [6,7], it could be expected that the motif involved does not differ significantly across species. We rationalised, therefore, that ExportPred could be applicable for prediction of exported proteins in the genus *Plasmodium*. To test whether the PEXEL export mechanism is also conserved across the phylum Apicomplexa, we used ExportPred to make predictions on the two other completely sequenced and annotated apicomplexan species, *Cryptosporidium hominis *[20] and *Theileria parva*, and also on a preliminary sequence of *Toxoplasma gondii*. Examination of the small number of positive predictions (*Cryptosporidium*, 20; *Theileria*, 9 (Additional data file 4); *Toxoplasma*, 36 (data not shown)) indicated that in each species only a few proteins were neither conserved across eukaryotes or were orthologous to a *Plasmodium *protein lacking an export motif. In addition, none of the predicted sequences from *Cryptosporidium*, *Theileria *or *Toxoplasma *form paralogous clusters, as could be expected for proteins exposed to the host immune system. We concluded, therefore, that PEXEL-mediated export into the host cell is most likely specific to the genus *Plasmodium*.

**Figure 4:**
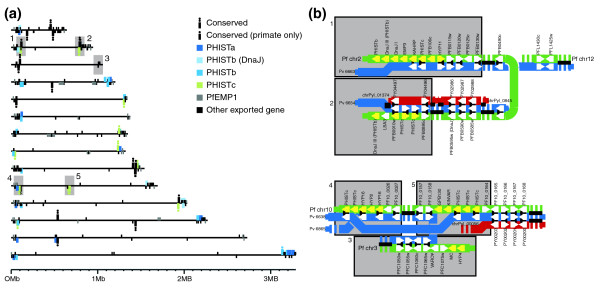
Chromosomal location of exported *P. falciparum *proteins and synteny with *P. vivax *and *P. yoelii *contigs. **(a) **Map of 14 *P. falciparum *chromosomes showing the location of exported genes conserved in *Plasmodium*, or only in the *P. vivax *and *P. falciparum *lineages. Location of var genes is shown for reference purposes, and PHIST genes are coloured. Shaded loci correspond to regions of synteny depicted in **(b)**: 5 syntenic loci on *P. falciparum *chromosomes 2, 3 and 10 containing conserved exported genes. *P. falciparum *chromosomes are shown in green, *P. vivax *contigs in blue, and *P. yoelii *contigs in red. Gene positions are represented by arrows; yellow arrows on *P. falciparum *chromosomes represent exported genes. Locations of *P. vivax *genes are inferred by reciprocal best hits homology, or where less stringent homology is augmented by parsimonious strand information and neighbourhood synteny. *P. yoelli *genes and orthology are as extracted from PlasmoDB [34]. Locus 1 on chromosome 2 shows that synteny begins with incomplete homology between KAHRP. Loci 1, 2 and 5 show conservation of PHISTc family members, but not of PHISTb. Loci 4 and 5 suggest an explanation for clusters of exported genes in central locations on *P. falciparum *chromosomes. In both cases exported genes exist at the ends of extremely long contigs, suggesting that they are subtelomerically located whereas the syntenic *P. falciparum *genes in locus 5 are centrally located. Locus 5 also demonstrates the breakdown in synteny at the location of PHISTc genes in *P. yoelli*.

We investigated the potential presence of a 'core set' by performing a reciprocal BLAST search for ortholog clusters of the *Plasmodium *and *Cryptosporidium *sequence sets. Out of 6,396 ortholog clusters, 277 had at least one ortholog with a predicted PEXEL score of ≥ 4.3. We further reduced this number by requiring that all members of the cluster had either a positive ExportPred score or a correctly aligned PEXEL motif but lacked a positive prediction due to a missing signal sequence (in case the first exon of the associated gene model was misannotated), and by ensuring that the motif was not contained in a functional domain. This resulted in 36 ortholog clusters conserved between at least two studied species in the genus *Plasmodium *(Table 3). None of these clusters had an ortholog in *Cryptosporidium hominis*, and we could also not find any in the other apicomplexan genomes of *Toxoplasma gondii *and *Theileria parva*. The *P. falciparum *'core' complement follows the expression pattern of exported proteins as described previously [6], with a peak in late schizonts, merozoite and ring stages consistent with a role in erythrocyte remodeling. While all 36 genes share an ortholog between *P. falciparum *and *P. vivax*, only 10 are also present in the genome of malaria parasites of the *P. berghei *lineage. Twenty-two belong to novel gene families identified in the course of this study: sixteen genes belong to the PHISTc subfamily, four belong to HYP11 and two to HYP16. In addition, one conserved gene, PFE0055c, encodes a DnaJ protein, and *PFB0915w *encodes a previously described liver stage antigen, LSA-3 [21]. The genes are clustered in the subtelomeric regions of *P. falciparum *chromosomes 1, 2, 3, 9, 10 and 11, respectively (Figure 4a). An alignment of the subtelomeric regions on chromosome 2 with *P. vivax *contigs demonstrates that synteny breaks down around *PFB0100c *(Figure 4b), which encodes KAHRP. KAHRP is the major structural knob component and chromosome breaks in the *KAHRP *locus occur frequently in *P. falciparum *and result in reduced cytoadherence [22-24]. On the other subtelomeric end of chromosome 2, synteny between *P. falciparum *and *P. vivax *and *P. yoelii *breaks down just upstream of a gene encoding an exported DnaJ protein (PFB0920w). We also investigated *P. falciparum *chromosome 10, since it contains 7 conserved genes encoding putatively exported proteins. Interestingly, the conserved subtelomeric cluster (except the most telomeric PHISTc gene PF10_0021) is syntenic with a large *P. vivax *contig that otherwise maps to the subtelomeric region of *P. falciparum *chromosome 3. This apparent chromosomal rearrangement event inserted approximately 50 genes between PF10_0021 and PF10_0163 (another PHISTc) and, therefore, moved this part of the conserved cluster towards the centromere in *P. falciparum*.

In addition to orthologous clustering, we examined exported proteins in other *Plasmodium *species where gene predictions were available. The close evolutionary relationship between the three studied species of the *P. berghei *lineage (*P. yoelli*,*P. berghei *and *P. chabaudi*) and between the two species of the *P. vivax *lineage (*P. vivax *and *P. knowlesi*) motivated our decision to combine predictions from individual species into 'hybrid' exportomes [25]. The predicted hybrid exportomes are considerably smaller than the *P. falciparum *complement (Figure 3e and Additional data file 3). Most significantly, both hybrid exportomes appear to contain only one large (>ten paralogs) lineage-specific family of exported proteins, an as yet unidentified one in the *P. vivax*/*P. knowlesi *cluster and the pyst-b family in the *P. berghei *lineage [26]. Intriguingly, members of the well-described *P. vivax *vir family [27] (except the virD subtype) of surface antigens and the related yir/bir/cir family of the *P. berghei *lineage lack a discernible PEXEL motif.

### Lineage-specific radiation of conserved proteins

Comparison of the *P. falciparum *exportome with the combined exportomes of the three species from the *P. berghei *lineage and the two studied from the *P. vivax *lineage clearly indicates an expansion of exported proteins in the *P. falciparum *lineage. This is reflected in the large number of *P. falciparum *gene families that encode exported proteins. While some gene families appear to be unique to this species (and the closely related *P. reichenowi*), others are present in the other two lineages either as single copy genes, or, in a few cases (for example PHIST, HYP11, HYP16) as an already radiated gene family.

### The FIKK kinases: a novel family of exported *P. falciparum *proteins

Recently, the identification of a novel class of putative protein kinases has been reported, termed FIKK kinases, in the phylum Apicomplexa [18,19]. The FIKK kinases are expanded in the *P. falciparum *lineage with at least 6 paralogs in (the incompletely sequenced genome of) *P. reichenowi *and 20 in *P. falciparum *(strain 3D7). Although enzymatic activity has not been demonstrated, the presence of most of the conserved residues of the catalytic domain suggests they are functional protein kinases [19]. The 20 *P. falciparum *paralogs all contain a PEXEL motif following an amino-terminal signal sequence (encoded in a short first exon) [18]. In contrast, the single orthologs from species of the *P. berghei *and *P. vivax *lineages lack the first exon encoding the signal sequence, as well as the PEXEL motif. Surprisingly, we found an additional FIKK paralog lacking the first exon and a PEXEL motif in the genome of another *P. falciparum *strain, a Ghanian isolate that is being sequenced at the Sanger centre (currently eightfold coverage) [28]. This suggests that radiation of the FIKK family was the result of PEXEL conversion of a sequence arising from an ancient gene duplication event in the *P. falciparum *lineage, with subsequent loss of the ancestral version occurring recently in the 3D7 strain.

### A novel family of exported proteins shared between two malaria lineages

**Figure 5:**
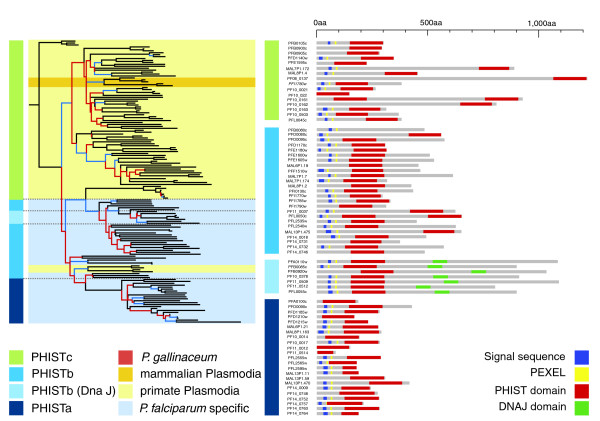
PHIST phylogeny and domain map. The PHIST tree (tree topology determined by bootstrapped neighbour joining based upon pairwise distances between instances of the PHIST domain; branch lengths assigned with least squares error minimisation; branches with <50% bootstrap support in red, 50% to 75% in blue, >75% in black) demonstrates conservation of the domain across Plasmodia. Colours indicate subfamilies (as determined by recognition by subfamily HMMs) and species conservation. Domain diagrams indicate organisational differences between subfamilies. The PHIST domain is carboxy-terminal in the PHISTc subfamily, regardless of length. In the PHISTa and b subfamilies a domain position of 100 to 200 amino acids from the amino-terminal methionine appears to be a general rule. In all the DnaJ containing members of the PHISTb subfamily, the DnaJ domain is carboxy-terminal to the PHIST domain. Members of the PHISTa subfamiliy are the shortest members of the PHIST family and are, as a whole, the most divergent and appear in a number of instances to be truncated. Note that PHIST domain representation is based on the annotated PlasmoDB [34] sequence, which in some cases lacks the first exon (for example, PFB0905c).

As depicted in Table 3, 16 out of 36 genes shared between the two *Plasmodium *lineages that infect primates belong to a novel gene family we have named PHIST. Initial alignments indicate the presence of a conserved domain of approximately 150 amino acids in length. We used a collection of HMMs constructed from subgroupings of domain sequences to the different *Plasmodium *species and identified 71 paralogs in *P. falciparum*, 39 in *P. vivax*, 27 in *P. knowlesi*, 3 in *P. gallinaceum *and 1 each in *P. yoelli*, *P. berghei *and *P. chabaudi *(Figure 5). The domain itself is predicted to consist of four consecutive alpha helices and does not appear similar to any known protein sequence (based upon searches of the NR protein database with PHIST HMMs) or structure (as determined by structural modelling based upon the structures of the top five weak similarities detected by the structure threading algorithm Fugue [29]). Interestingly, PHIST domains cluster into three distinct subgroups (termed PHISTa, PHISTb and PHISTc), which are distinguished by the presence and position of several conserved tryptophans. In addition, the three subtypes show different overall structures: PHISTa proteins are very short and consist only of a signal sequence, an export motif and the PHIST domain; PHISTb proteins show more length variability in the carboxy-terminal portion following the PHIST domain, and a subset of seven PHISTb proteins, including the well-characterised vaccine candidate RESA (Ring-infected surface antigen [30,31]), contains an additional DnaJ domain; PHISTc proteins are the most diverse group and the only one that radiated before the separation of the *P. falciparum *and *P. vivax *lineages (Figure 5). The genes encoding the three subfamilies are located in subtelomeric regions of all chromosomes, except chromosome 3. Microarray data from *P. falciparum *strain 3D7 indicate the typical expression pattern of exported proteins for PHISTb and PHISTc; a peak during schizogony and after invasion in the ring stage parasites. In contrast, PHISTa genes are generally not detectably transcribed: only two genes show significant transcription levels.

### Expansion of exported J-proteins in *P. falciparum*

**Figure 6:**
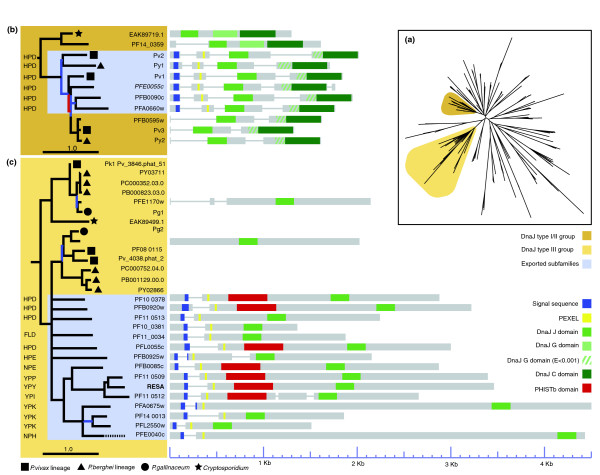
DNAJ phylogeny and gene/domain models. **(a) **The inset shows the complete tree of DnaJ domain sequences extracted from different *Plasmodium *species and *C. hominis*. Subtrees containing domains from exported **(b) **type I and **(c) **type III proteins are highlighted. DnaJ subtrees (type I, bootstrapped neighbour joining on full sequences; type III, TREE-PUZZLE on full sequences; branches with <50% bootstrap support in red, 50% to 75% in blue, >75% in black) show export (blue boxes) and core motif variations. Sequences not belonging to *P. falciparum *are marked with symbols to indicate the species group from which they arise. PF14_0359 has orthologs in other Plasmodia (not shown). Positions of signal sequence and PEXEL as determined by ExportPred, and PHIST and DnaJ domains as determined by HMM search, are overlaid on gene models. Although the large number of family members tends to decrease bootstrap support for individual branches, congruence with the three PHIST subfamilies as defined by HMM profiles lends additional support to the overall topology of the tree.

Apart from RESA, several other *P. falciparum *sequences with homology to the RESA J-domain were identified in an early genomic survey [32]. In eukaryotic cells, proteins carrying a J-domain act as co-chaperones, regulating the activity of 70 kDa heat-shock proteins (HSP70s). Three classes of J-proteins (also called HSP40s) are distinguished by the presence and nature of the three domains originally identified in the bacterial protein DnaJ. Type I members contain all three domains: the J-domain (including a highly conserved HPD motif essential for interaction of the protein with HSP70), a glycine-rich domain and a carboxy-terminal zinc-finger domain. Type II members lack the zinc-finger domain. Type III members contain the J-domain only. While type I and type II proteins act as true chaperones, interacting with substrate proteins as well as regulating Hsp70 activity, the function of type III members is less clear [33]. Since our ExportPred output (score ≥ 4.3) contained several proteins annotated on PlasmoDB [34] as putative heat shock proteins (due to the presence of a J-domain), we used the PFAM DnaJ domain for an HMM search in PlasmoDB [34]. The HMM analysis identified 43 *P. falciparum *proteins with an amino-terminal DnaJ domain, of which 19 are predicted to be exported. In comparison, the apicomplexan parasite *C. hominis *genome encodes 24 proteins with DnaJ domains, which is similar to the complement in the yeast *Saccharomyces cerevisiae *(22 plus 3 J-like proteins [33]). A phylogenetic analysis of all J-proteins from *S. cerevisiae*, *C. hominis *and different *Plasmodium *species indicated two clusters with a lineage-specific expansion of DnaJ proteins (Figure 6a). One cluster represents the radiation of type I or type II (no clear prediction of the central G domain) DnaJ proteins before the divergence of the *P. berghei *lineage from the *P. falciparum *and *P. vivax *lineages (Figure 6b). One *P. yoelii*, two *P. vivax *and three *P. falciparum *genes share the same four-exon structure with a signal sequence encoded in exon 1 and a predicted PEXEL motif encoded in exon 2. In addition, the amino-terminal J domain contains the conserved HPD motif, which is an essential feature of known J-domains in enabling the regulation of Hsp70 activity [33]. A second cluster, including RESA, represents expansion of type III DnaJ proteins in *P. falciparum *only (Figure 6c). These 15 genes encode highly divergent proteins with various amino acid differences in the crucial HPD motif. *S. cerevisiae *has three type III DnaJ proteins with similar mutations in the HPD domain (for example, H to Y in position 1, D to E in position 3), which were consequently classified as J-like proteins [33]. Since the function of the three yeast proteins is unknown, it remains to be determined whether the exported *P. falciparum *proteins with mutations in the HPD domain are capable of stimulating ATP hydrolysis of HSP70 and, therefore, whether they are functional DnaJ proteins. As mentioned earlier, seven proteins of the PHIST protein family are also part of these unusual DnaJ proteins due to the presence of a carboxy-terminal J-domain (Figures 5 and 6b).

## Discussion

Identification of a conserved signal mediating protein export from *P. falciparum *into the host red blood cell has facilitated the systematic identification of the 'exportomes' of malaria parasites. One goal of this study was the development of a program to characterise the exportomes of the three malaria parasites infecting rodents, *P. yoelii*, *P. berghei *and *P. chabaudi*, and the two malaria parasites infecting primates, *P. vivax *and *P. knowlesi*; this has been used to determine the 'core' exportome of the genus *Plasmodium*. Interestingly, we have identified novel gene families that have radiated to differing extents in these parasites. It is likely that the conserved core exported proteins are important in the remodeling of the host erythrocyte for the different malaria species, while the genes that are specific for the much larger exportome of *P. falciparum *probably encode proteins that are directly or indirectly involved in the different properties of this parasite.

### ExportPred: prediction of *Plasmodium *exportomes

HMMs [9,35], used here to develop ExportPred for the prediction of the *Plasmodium *exportome, have been successfully applied to a number of problems in sequence analysis, including signal sequence prediction (SignalP [8], Phobius [36]), transmembrane domain prediction (TMHMM [37]) and secondary structure prediction. In the majority of these cases, the presence of a significant volume of biochemically verified data is offset by the weakness of the signal they aim to detect. Although the situation for *Plasmodium *export prediction is somewhat reversed - paucity of verified data is made up for by the strength of the signal - the PEXEL motif provides an opportunity for accurate algorithmic prediction. GHMMs extend HMMs by augmenting states with explicit length distributions, allowing the use of a smaller number of states to capture a more general set of features at the expense of an increase in computational complexity and the lack of a procedure for automated training. Features such as the periodicity observed in the signal sequence length are well captured by a single state in a GHMM, whereas to represent the observed set of lengths in a HMM would require a complex group of states with a predetermined topology. The choice of a GHMM based architecture for PEXEL prediction improves upon rule-based methods by utilising a probabilistic framework, which captures all the observed sequence features of PEXEL containing proteins simultaneously, while not excessively increasing complexity. Some differences are observed between previous exportome predictions and those of ExportPred. In one study the exportome had been hand curated and, therefore, a large proportion of the predictions unique to it can be ascribed to cases in which an incorrect gene model was reannotated, generally to correct a missing or incorrect first exon [6]. Given the similar performance of the motif published in another study to the ExportPred framework [7] (Table 1) coupled with the discrepancy in predictions, we surmise that their exportome is unnecessarily conservative, and stems to some degree from the reliance on positive SignalP prediction, which does not allow for recessed signal peptides, a common feature of exported proteins.

The vast majority of the predicted exportome (Figure 3a) is encoded by genes that are located in the subtelomeric regions of most *P. falciparum *chromosomes, and that tend to have a two-exon structure with the signal sequence in the first exon and the PEXEL motif at the beginning of the second exon. In addition, a large proportion of exported proteins encoded by genes with more than two exons show the same configuration for the first and second exons and of the 36 exported proteins conserved across Plasmodia, 33 are encoded by 2 exon genes (Table 3). Two competing models may explain this surprising finding. The two-exon structure may represent the ancestral status of genes encoding exported proteins, and consequently the present situation is the result of a large radiation from a single ancestral gene. Alternatively, this specific gene structure could be the consequence of a recombination mechanism, which inserts either the first exon only or, more likely, the whole 'pre-sequence' encoding the signal sequence and the PEXEL motif into existing genes. Pre-sequence acquisition has been described for chloroplast and mitochondria-derived genes transferred to and encoded in the nucleus, and which, therefore, are forced to acquire pre-sequence encoding exons to target the corresponding proteins back to their respective organelle [38-40]. Considering the case of the exported FIKK kinases and DnaJ proteins, which are both encoded by genes with this typical structure and which have apparently evolved via an initial gene duplication of a one-exon gene, pre-sequence acquisition by exon-shuffling is the most parsimonious mechanism. A similar model, termed semi-exon shuffling, has recently been proposed for the acquisition of plastid-targeting transit peptides in diatoms [40]. Interestingly, a recent study found an excess of phase 1 introns around signal sequence cleavage sites in human genes [41], while the introns in genes encoding exported *P. falciparum *proteins are predominantly in phase 0.

### The 'core' exportome: novel families and conserved loci

The ExportPred output from a whole genome analysis of *C. hominis*, and the lack of conserved exported proteins encoded in other apicomplexan genomes such as *T. gondii *and *T. parva *(T Sargeant, unpublished observation), indicate that the evolution of the PEXEL motif, in its described form, is specific to the *Plasmodium *genus. Recently, a connection has been drawn between the *Plasmodium *PEXEL motif and a short amino-terminal sequence (RxLR) observed in three effector proteins of the oomycete plant pathogen *Phytophthora *[42]. Thus far, no experimental evidence supporting its function in *Phytophthora *exists and the lack of any similar motif in organisms much more closely related to the genus *Plasmodium *makes it unlikely that the *Phytophthora *motif is evolutionarily related to the PEXEL motif.

**Figure 7:**
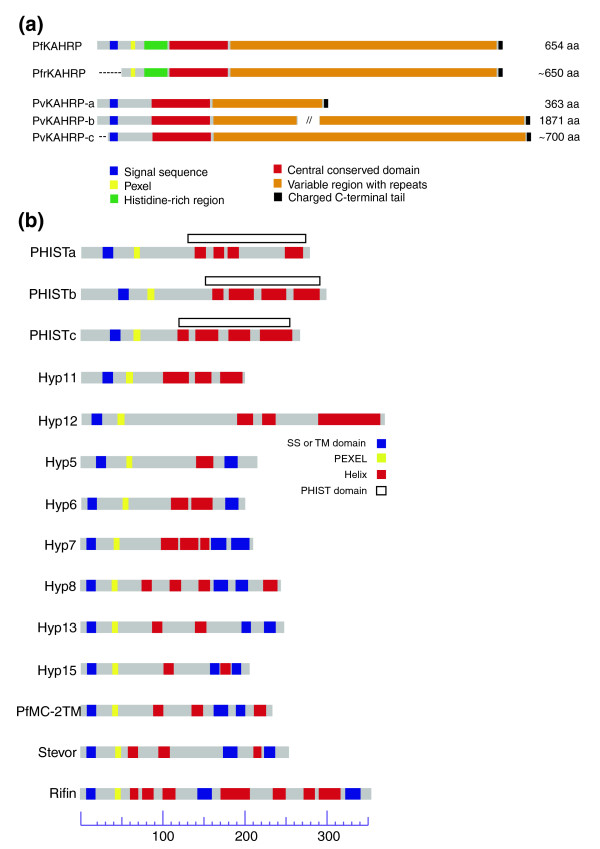
Plasmodium KAHRP orthologs and structural similarities of exported proteins. **(a) **Structural alignment of *P. falciparum *KAHRP and orthologs from *P. fragile *and *P. vivax*. Note that *Pfr*KAHRP and *Pv*KAHRP-c represent incomplete sequences (amino terminus represented as dashed line). **(b) **Structural representation of *P. falciparum *exported protein families. Secondary structure prediction was performed using the consensus method Jnet [84,85].

Although the export motif is conserved in the majority of orthologous groups, there are a few exceptions where the PEXEL has apparently been acquired in a lineage-specific manner. The radiation of FIKK kinases and two subfamilies of putative a/b hydrolases (our unpublished observation) in the *P. falciparum *lineage suggest an initial gene duplication event of a conserved, non-secreted protein specifically in this lineage. Interestingly, we have identified predicted proteins in *P. vivax *and *P. knowlesi *with high homology to the central region of KAHRP. The KAHRP amino terminus consists of a signal sequence and a PEXEL motif for entry into the secretory pathway and subsequent export into the host cell (Figure 7a). A histidine-rich region (His-domain) downstream of the PEXEL motif and two charged repeat regions in the carboxyl terminus of the protein are implicated in Maurer's clefts binding and association of KAHRP with PfEMP1, respectively [1,43]. In contrast, the putative *P. vivax *and *P. knowlesi *KAHRP orthologs contain neither a discernible PEXEL motif or His-domain, and the repeat region is much shorter and of different amino acid composition. However, a KAHRP ortholog from *P. fragile *with 97% identity to PfKAHRP across the whole length has recently been deposited to GenBank (accession number CAB96390) [44]. The lack of a non-exported KAHRP ancestor in *P. reichenowi *and *P. falciparum *suggests a lineage-specific conversion of a common KAHRP ancestor into an exported protein associated with the knob structure, or gene loss after an initial gene duplication event. It is interesting to note that knob formation and cytoadherence have been demonstrated in *P. fragile *but not in *P. vivax *or *P. knowlesi *[45]. In *P. falciparum*, it has recently been shown that absence of knobs due to deletion on chromosome 2 results in reduced surface expression of PfEMP1 and, therefore, reduced cytoadherence [46]. However, it remains to be shown which of the four genes apart from *rif *and *var *genes (KAHRP itself, PfEMP3, the two DnaJ typeI/II gene products PFB0085c and PFB0090c, or the PHISTc paralog PFB0080c) on the deleted chromosome end are responsible for this effect.

Radiation of DnaJ proteins in *Plasmodium *appears to have occurred twice independently. Our presumption is that initial gene duplication in a common ancestor of all three parasite lineages generated one DnaJ type I/II protein exported into the host erythrocyte. Duplications occurring after the divergence of the *P. berghei *lineage gave rise to a second copy in the *P. falciparum *and *P. vivax *lineages and a third copy in *P. falciparum *- though whether one of these duplications occurred before *P. falciparum *speciation is not clear. Although orthology is not able to be determined based upon protein sequence, indications from synteny observations suggest PFE0055c to be the ancestral exported type I DnaJ protein. Expansion of exported DnaJ type III proteins (including those with an additional PHIST domain), on the other hand, has occurred in *P. falciparum *only. Although the strict conservation of the crucial HPD motif in all type I/II and four type III proteins predicts them to be functional co-chaperones of HSP70, no such *P. falciparum *protein has a putative export motif. The *P. falciparum *genome encodes six proteins with HSP70 domains: two appear to be cytoplasmic proteins (PF08_0054, chr7_000093.glm_5), one contains a predicted mitochondrial transit peptide (PF11_0351), two contain carboxy-terminal ER retrieval sequences (including PfBIP [47]; PFI0875w, MAL13P1.540), and one is the *P. falciparum *ortholog (PF07_0033) to the cytoplasmic HSP105. A recent report demonstrated the recruitment and ATP-dependent function of host-derived HSP70 on the surface of infected red blood cells [48]. Moreover, the chaperone co-migrated in a large complex with KAHRP, suggesting localisation to the knobs. It is possible, therefore, that exported DnaJ proteins function as co-chaperones and with host HSP70 are involved in refolding highly complex molecules such as the virulence factor PfEMP1. Interestingly, a subgroup of DnaJ type III proteins also contains a PHISTb domain. The proteins in this subgroup, which includes RESA, contain a slightly relaxed PEXEL motif, RxLxxE (which is not currently modelled by ExportPred) and an unusually recessed hydrophobic signal sequence, which SignalP does not predict to be proteolytically cleaved. The expression of RESA (and all the other paralogs (see Additional data file 2)) peaks during late schizogony, and it has been demonstrated that RESA is resident in the dense granules before accumulating in the parasitophorous vacuole and being translocated into the host cell after invasion. In light of this, it is possible that RESA, and the other paralogs, are translocated into the ER as a signal-anchored protein and subsequently trafficked to dense granules. Cleavage of the anchor presumably then occurs in the dense granules or after entry into the parasitophorus vacuole, allowing recognition of the PEXEL by the parasitophorus vacuole membrane translocation machinery [49].

The PHIST protein family, identified for the first time in this work, is organised into three subfamilies, of which PHISTc is entirely shared between the *P. falciparum *and *P. vivax *lineages and, in single copy form, in the three species of the *P. berghei *lineage. The PHISTb subfamily appears to contain only two members in *P. vivax *and *P. knowlesi*, but has radiated extensively in *P. falciparum*. The PHISTb subfamily contains a *P. falciparum *specific subgroup including RESA that is distinguished by the additional presence of a DnaJ domain. In contrast to PHISTb and PHISTc, PHISTa is entirely *P. falciparum *specific. It is difficult to assign possible functions to PHIST proteins as none of the members of this family have previously been described (apart from the unusual RESA antigen), and the helical structure of the prototypical PHIST domain appears dissimilar to any known structure. It is clear, however, that PHISTc performs a function common to the genus *Plasmodium*, but which is expanded in the *P. falciparum *and *P. vivax *lineages, while the function of PHISTa is specific to *P. falciparum*. A recent yeast-two hybrid based analysis of a global protein interaction network in *P. falciparum *indicates a PHISTb and PHISTc paralog (PFD1140w and MAL7P1.7, respectively) plus two PHISTb paralogs with a DNAJ domain (RESA and PF11_0509) being part of a interaction network with skeleton-binding protein [50] in the centre [51]. While most *PHISTb *and *PHISTc *genes show transcriptional peaks mainly in early asexual stages, three recently published microarray studies [52-54] indicate that a number of genes from these two subfamilies (plus most paralogs of the PfMC-2TM family, and an infected red blood cell membrane protein PIESP2 [55]) are specifically upregulated in early gametocyte stages (see Additional data file 2). Interestingly, the same PHISTb and PHISTc genes also appear upregulated in parasites directly extracted from infected patients compared to the culture-adapted 3D7 [56]. Genes of the *PHISTa *subgroup on the other hand appear transcriptionally silent in 3D7 (with the exception of the unusal *PFD0090c *and one other member, *PFL2565w*), and it is therefore possible that *PHISTa *genes are subject to mutually exclusive expression (as is the case for the *var *genes). Proteomic data show a consistent presence of PHIST proteins in parasite-infected erythrocyte membrane fractions [55,57,58], and the PHISTc paralog PFI1780w has been detected in detergent-resistant membrane fractions [59]. PHIST HMMs do not detect sequence homology with any non-*Plasmodium *protein in the NR database, and a sequence-structure homology search, followed by structure modelling using the best hits, also produced no acceptable results. As such, it is possible that this domain represents a novel protein fold specific to *Plasmodium*, although it should be noted that the weak homology between members of the PHIST family in *P. falciparum *alone suggests that any structural similarity is likely to be hard to detect on the basis of primary sequence. Finally, the topology of the PHIST tree is a strong indication for a closer phylogenetic relationship between the *P. falciparum *and the *P. vivax *lineage than either of those and the *P. berghei *lineage.

Apart from the conserved families, only a few genes encoding hypothetical exported proteins are conserved in *P. falciparum *and one or both of the two other studied lineages. Importantly, both single copy genes and paralogs from the three conserved families PHIST, HYP11 and HYP16 are predominantly clustered in the subtelomeric regions of *P. falciparum *chromosomes 2, 9 and 10. Completion of the whole genome sequencing and chromosomal mapping of *P. falciparum *[60] and *P. yoelii *[61] confirmed previous chromosomal mapping studies that indicated a high degree of synteny among different *Plasmodium *species. Apart from the subtelomeric regions, gene synteny and order appear to be conserved over large stretches between species within the *P. berghei *lineage, and to a lesser extent between *P. falciparum *and *P. vivax *[62,63]. On the other hand, it has been demonstrated that genes encoding variant surface antigens and exported proteins are located subtelomerically, and that these regions exhibit the highest genomic plasticity [6,64]. A comparison of chromosome 2 has shown that single nucleotide polymorphism (SNPs) between different *P. falciparum *isolates are clustered in the subtelomeric regions [65]. In this study, we have analysed the subtelomeric regions of chromosomes 2 and 10, which contain many of the conserved genes encoding exported proteins. Synteny between large *P. vivax *contigs and *P. falciparum *chromosome 2 breaks down where a large increase in SNPs between isolates has been reported [65]. In addition, one subtelomeric region of chromosome 10 appears to be the result of a lineage-specific chromosomal rearrangement with a subtelomeric portion of chromosome 3 (Figure 4b).

### Exported proteins: conserved structure - conserved function?

The clustering of exported *P. falciparum *proteins into paralogous groups revealed a large number of novel families, some of which are even conserved between different *Plasmodium *lineages (for example, Hyp11, Hyp16, PHIST). Strikingly, the majority of these proteins, including Rifin, Stevor, PfMC-2TM and PHIST, are between 250 and 300 amino acids in length and include mainly helical stretches (Figure 7b). Altogether, approximately 75% of all exported proteins (excluding PfEMP1) conform to this structure. Moreover, four novel protein families (Hyp7/8/13/15) as well as Rifin, Stevor and PfMC-2TM contain two transmembrane domains, which are separated by three (PfMC-2TM) to 170 amino acids (Rifin). Based on the presence of the two closely positioned transmembrane domains, a *P. falciparum*-specific superfamily encoded by subtelomeric multicopy gene families has been postulated [17]. In addition, another study suggested a common ancestry for the large antigenic families present in the three species of the *P. berghei *lineage (*yir/bir/cir*), *P. vivax *(*vir*) and *P. falciparum *(Rifin) based on their structural similarities [66]. In this context, it is interesting to note that unlike yir/bir/cir and vir proteins, which all lack an export motif, the virD subfamily paralogs show the same overall structure as Rifin, including the amino-terminal signal sequence and export motif (also predicted by ExportPred with a score ≥ 4.3). However, it remains to be shown whether virD is indeed the link between Rifin and the rest of this superfamily, that is, whether this postulated relationship is genuine. The presence of two (carboxy-terminal) transmembrane domains raises questions about the orientation and function of these proteins in their respective target membrane. It has been proposed that the highly variable portion flanked by these two hydrophobic stretches forms a loop, which is exposed to the cytosol (in the case of Maurer's clefts resident proteins) or the host environment (in the case of Rifin and probably Stevor) [17]. In general, the similarity in structure of these protein families suggests that they have either been derived from the same ancestral gene or, alternatively, that these similarities are the result of convergent evolution reflecting the structural constraints of proteins exported to the host cell.

## Conclusion

Synteny of conserved clusters between *P. vivax *and *P. falciparum *(and to a certain extent *P. yoelii*) demonstrates the presence of an ancestral set of exported proteins located on a few conserved loci present in a common ancestor. The fact that most exported proteins in the different exportomes share a common secondary structure with the few conserved ones (short, soluble, one or two carboxy-terminal transmembrane domains, multiple alpha helices) strongly suggests that these ancestral core proteins were the template for subsequent lineage- and species-specific radiations. Conservation of the core set of exported proteins in *Plasmodium *strongly suggests that they are important in remodeling of the parasite-infected erythrocyte. In contrast, the large set of proteins that appear to be unique to specific Plasmodia are likely to provide distinct functions for survival in host specific erythrocytes. It is interesting that *P. falciparum *appears to have a greatly expanded exportome compared to other Plasmodia and the most likely reason for this striking observation is the presence of PfEMP1 in *P. falciparum *that is trafficked to the surface of the infected erythrocyte. PfEMP1 is a complex multidomain protein and the presence of a family of putative co-chaperones containing DnaJ domains may be involved in the translocation process and correct conformation of this functionally important virulence protein. The identification of a conserved set of exported proteins in *Plasmodium *strongly supports the idea that these are functionally critical and are potential drug targets for the development of a novel class of drugs against this important infectious disease.

## Materials and methods

### Sequences

We extracted 170,743 protein sequences from *Plasmodium *and *Cryptosporidium *from PlasmoDB [34], GeneDB [67], the Sanger Centre [28] and GenBank [44]. *Cryptosporidium *sequences were used in this study both as an outgroup to the genus *Plasmodium *and also to determine whether the PEXEL motif represents an export signature conserved across Apicomplexa. The composition of this set is as follows: 5,334 annotated sequences and 17,679 automatic predictions for *P. falciparum *taken from the 2002 PlasmoDB [34] release; 37,501 *P. reichenowi *open reading frames (ORFs; >50 amino acids) extracted from the 11/03/04 Sanger Centre [28] contig set; 12,216 *P. berghei *sequences from PlasmoDB [34]; 14,896 unique *P. chabaudi *sequences from PlasmoDB [34]; 9,090 unique *P. yoelii *sequences from the union of Py17XNL_WholeGenome_Annotated_PEP and Pyl17XNL_2002.09.10_SeqCenterPredictions.fasta (PlasmoDB; now superseded); 47,406 *P. gallinaceum *ORFs (>50 amino acids) from the 23/09/04 Sanger Centre [28] contig set; 7,112 unique *P. knowlesi *sequences from the union of Pk_prot.fa (PlasmoDB [34]) and pkn_prots.db (Sanger Centre [28]); 10,832 *P. vivax *sequences (PlasmoDB [34]); 8,677 *Cryptosporidium *sequences extracted from GenBank [44].

### Training set

The training set of 166 sequences was taken from [6] and contained manually curated exported protein sequences encoded by genes with two exons, expressed early in the intra-erythrocytic cycle and/or during late schizogony. Of these, 23 have been directly validated, or have proteomic evidence for infected red blood cell (iRBC) localisation. A further 35 are paralogous to these 23. This set of sequences did not contain any members of the exported Rifin or Stevor families, which were used as a test set. For training the KLD path, all annotated PfEMP1 sequences from *P. falciparum *3D7 were used. We have produced a retrained version of ExportPred using this conservative subset; examination of the output of this retrained version revealed only negligible differences from the version trained on the full set of sequences.

### Simulated negative sets

Simulated negative sets were constructed using a second order Markov model trained on *P. falciparum *protein sequences. Acknowledging the possibility of contamination, complete single exon genes with a non-subtelomeric chromosomal location (>500 kilobases from a telomere) were used as a non-simulated negative set (PfNegative; 1,292 sequences). Simulated test sets containing signal sequences but no PEXEL were also created by four methods: using the second order Markov model (NoSS; 1,000 sequences); using the SignalP model to simulate signal sequences, concatenated with a tail simulated using the second order Markov model (SpSS; 1,000 sequences); using amino termini from real proteins with a predicted signal sequence, one exon, and a non-subtelomeric chromosomal location followed by a simulated tail (PfSS; 165 sequences); and using the ExportPred signal sequence model to simulate signal sequences, followed by a tail simulated using the second order Markov model (EpSS; 1,000 sequences).

### ExportPred

ExportPred utilises a GHMM to score protein sequences according to their likelihood of export. The architecture of the ExportPred GHMM (Figure 1a) consists of three paths. Two paths model, respectively, RLE (RxLxE/Q) PEXELs (of which Rifins and Stevors are members) and KLD (KxLxD) PEXELs (of which PfEMP1 is the only described example) and the third provides a background model. A simple model for signal peptides was selected, capturing only the observed leader and hydrophobic signal sequence length distributions and amino acid composition. RLE type PEXELs were thus modelled by five states corresponding to the leader, the hydrophobic stretch of the signal sequence, spacer, the RxLxE/Q motif and the carboxy-terminal portion of the protein. KLD type PEXELs consist of a shorter leader sequence, followed immediately by an instance of a KxLxD motif, which is more highly conserved than the RxLxE/Q motif. It is hypothesised that the transmembrane domain immediately amino-terminal of the acidic terminal sequence (ATS) acts in ER targeting, and has, therefore, also been included in the model. The null model consists of a single state modelling the average length and amino acid composition of the *P. falciparum *proteome. All three paths are tied together by a state representing the amino-terminal methionine, and transition probabilities were chosen to reflect our prior belief concerning the relative abundances of proteins in the three captured classes. An RLE (or KLD) score is derived by taking the log ratio of the joint probability of the sequence and the final state being RLE-tail over the joint probability of the sequence and the final state being NULL-tail.



Due to the lack of well annotated and validated training data, as well as the lack of parameter estimation procedures for GHMMs, all model parameters for ExportPred were derived empirically. Amino acid distributions for the five positions of the RxLxE/Q and KxLxD motifs, as well as for the amino acid positions immediately preceding and following, were derived from the multiple alignment presented in [6]. Hydrophobic regions were modelled initially by an amino acid composition extracted from the hydrophobic stretches of the training sequences using a windowed approach, and with a length distribution allowing, with equal probability, any length between 10 and 25 residues. All other states were modelled with background amino acid compositions and approximate geometric length distributions. Using this initial model, residues in the sequences of the training set were classified according to the most likely state path for the sequence, and these classifications were then used to update the length and emission distributions of the model states. Length distributions for non-geometric states were smoothed using a gaussian kernel [68]. A kernel bandwidth of 1.0 was used for all states except for the RLE leader, where a bandwidth of 2.0 was used due to the greater variability in observed lengths. Variants of the ExportPred model were also produced that replaced the signal sequence or PEXEL portion of the model with the signal peptide model from SignalP (with the addition of a more flexible leader state to allow for recessed signal peptides) and/or the 11 residue motif described in [7], respectively. ROC curves [69] for the standard ExportPred model are shown in Figure 1b.

To compute a score threshold, we used the training set and the PfNegative set for positives and negatives, respectively, and, after correcting for our prior belief that approximately 10% of the *P. falciparum *is exported, generated a curve describing false discovery rate (FDR) as a function of score (Figure 1c). From this curve, a score threshold of 4.3 was selected, corresponding to a 5% FDR [70], meaning that we expect 5% of the resulting set of ExportPred predictions above this threshold to be false positives. A 5% FDR corresponds in this case to a false positive rate for individual sequences of approximately 0.5%. A threshold of 0.0 for the KLD path correctly identifies all *var *genes with a zero false positive rate. ExportPred is implemented in C++, and has been successfully compiled using recent versions of g++ on Linux and OSX. Source code is available on request.

### Compilation of the *P. falciparum *exportome

*P. falciparum *automated predictions and annotations were scored using ExportPred. Automated predictions and annotated genes were grouped where their chromosomal locations were congruent. Ties in which an automated prediction overlapped more than one annotation were broken by selecting the annotation with the greatest sequence overlap. To address the problem of misannotation of first exons, the gene model with the highest PEXEL score was selected from each group as a representative.

### Ortholog clusters

Ortholog pairs were determined from an all by all BLAST comparison (ignoring pairwise comparisons within a species) of the *Plasmodium *and *Cryptosporidium *sequence sets using the reciprocal best hit method [71,72]. Ortholog pairs in which one or both sequences had a positive PEXEL prediction were selected. Sequences were then grouped based upon these pairwise orthology relationships. Clusters were chosen such that all members were connected to all other members by orthology relationships either directly, or through a connected chain of orthology relationships (for example, inferring orthology between sequences in species A and C, where the sequence from species A is orthologous to a sequence from species B, which is also orthologous to the sequence in species C). Sequences in each cluster were aligned using MUSCLE [73].

### Clustering of *P. falciparum *protein sequence into families

Pairs of *P. falciparum *sequences were selected from the all by all comparison where there existed between the two sequences a reciprocal pair of BLAST hits with an E-value <10^-5 ^and with subject residues aligned to at least 50% of query residues, and where at least one of the pair was predicted to be exported. Connected subgraphs were selected to produce family groups in a manner analogous to that described above for ortholog clusters. Multiple alignments of family groups were constructed with MUSCLE and inspected to verify clustering. A number of already annotated families (Rifins [5,15], Stevors [16], PfMC-2TM proteins [17], FIKK kinases [18,19]) were checked to ensure that whole families had been retrieved by this method. Families for which no annotation was available for any members were assigned a HYP*n *(for HYPothetical family number *n*) temporary identifier.

### Determination of *P. falciparum *exportome expression patterns

Raw Affymetrix expression data from [14,52] were reanalysed using the RMA [74] and GCRMA [75] algorithms. For GCRMA, we used only the probe affinities in the background correction step, as mismatch probes were not available for this array. The analysis was carried out using the Bioconductor packages affy and gcrma [76]. For all stages, the maximum expression from sorbitol and heat treatments was chosen. Additionally, early and late stages of ring, trophozoite and schizont stages were summarised by maximisation. A probe intensity of 5 was taken as a conservative estimate of expression.

### Hybrid exportomes for the *P. berghei *and the *P. vivax *lineage

Proteins from *P. yoelli*, *P. berghei*, *P. chabaudi*, *P. vivax *and *P. knowlesi *predicted to be exported by ExportPred were curated to remove false positives caused by poor gene models. Each set was BLASTed against ORFs from genomes of the species represented in the set. Pairs of sequences with homology (<10^-5^) to similar sets of ORFs (determined by requiring that at least 50% of hits to ORFs by either sequence were shared by both) were clustered to simultaneously detect putative orthologs and families.

### DnaJ domain phylogeny

Sequences of DnaJ domains, including 10 amino acids amino- and carboxy-terminal to the domain were extracted from *Plasmodium *and *Cryptosporidium *sequence sets using hmmsearch (from the hmmer [35] package) with DnaJ HMM (PFAM accession: PF00226.16) and e-value cut off of 0.01 and aligned using hmmalign. Bootstrapped neighbour joining was used to choose a tree topology, and branch lengths were assigned using least squares error minimisation. Two subtrees were noted to contain sequences from *P. falciparum *proteins predicted to be exported. These subtrees were extracted, ensuring that one *C. hominis *sequence remained as an outgroup. Full length sequences were realigned using MUSCLE, and the resulting multiple alignment used to construct new trees using both neighbour joining and maximum likelihood puzzling (using TREE-PUZZLE [77]). TREE-PUZZLE was preferred over neighbour joining for small phylogenies, where it was computationally tractible and where neighbour joining did not produce meaningful results. For the exported DnaJ type I subtree, it was necessary to manually correct gene models for the three *P. vivax *and two *P. yoelii *sequences used. Secondary structure of individual domain instances was predicted using PSIPRED [78], from which a consensus structure prediction was derived for the multiple alignment.

### Bioinformatic analysis of the PHIST family

The PHIST family was initially detected as a single family (with the temporary identifier HYP3) by the family clustering algorithm described. From this initial group, consisting of 44 members, a multiple alignment was created using MUSCLE, and the conserved portion (which corresponds to a domain predicted by PSIPRED to consist of 4 α-helices separated by short coils) was selected to build a HMM using hmmbuild. Observations based upon the content of the PEXEL motif and upon phylogenetic analysis led us to further divide this group into a- and b- subfamilies, and HMMs covering the same conserved stretch were built for these two subfamilies. The three resulting HMMs were used to search the *P. falciparum *proteome, expanding the family membership. Any new sequence matching the superfamily HMM but not either of the a- and b-subfamily HMMs was assigned to a new c-subfamily. A HMM was built for the c-subfamily and used to expand its membership. Once complete lists of sequences in the three subfamilies were obtained, the three subfamily HMMs were recreated utilising sequence from all their respective members. Limited cross-recognition was observed between the b- and c-subfamily HMMs, and no cross-recognition was observed between the a-subfamily HMM and either of the b- and c-subfamily HMMs. The three subfamily HMMs were used to search the combined *Plasmodium *and *Cryptosporidium *database previously described, as well as the National Center for Biotechnology Information (NCBI) nr database. No significant hits existed outside the *Plasmodium *genus. Domains from all sequenced *Plasmodium *species were aligned using MUSCLE and used to create a bootstrapped neighbour joining tree. Branch lengths were assigned using least squares error minimisation, and *P. gallinaceum *sequences were used as an outgroup.

### Generation and analysis of GFP fusion proteins

Sequences encoding the amino-terminal portion of PFI1755c, PFE0055c, PFI1780w, PFE0360c, PF10_0321, PF14_0607 and PFE0355c were amplified from cDNA of an asynchronous *P. falciparum *culture, strain 3D7. Each of the sequences encodes the native amino terminus of the protein including the signal sequence, the predicted PEXEL motif plus an additional 11 amino acids downstream to allow for proper folding of GFP [10]. PCR products were subcloned (for primers see Additional data file 3) into the pENTR/D TOPO vector as part of the MultiSite Gateway™ cloning strategy (Invitrogen, Carlsbad, CA, USA), which has recently been adapted for *P. falciparum *expression vectors [79]. Expression vectors were generated by recombination of the destination vector pCHDR-3/4 with the three entry clones PfHSP86 5'-pENTR4/1, eYFP-pENTR2/3 and the gene-specific pENTR/D TOPO vector (for details of vector construction see [79]). Expression vectors were transfected into the *P. falciparum *line 3D7 and selected on 10 nM of the antifolate drug WR99210 as described previously [80]. Transgenic parasites were cultured under standard conditions [81] and analysed using a Zeiss Axioskop fluorescence microscope (Zeiss, Jena, Germany).

## Additional data files

The following additional data are available with the online version of this paper. Additional data file 1 is a table listing primers used for generation of *P. falciparum *expression vectors. Additional data file 2 is a table of the *P. falciparum *exportome. Additional data file 3 is a table of the exportomes of the *P. berghei *and *P. vivax *lineages. Additional data file 4 is a table of the ExportPred output from *C. hominis *and *T. parva*.

## Supplementary Material

Additional data file 1 Primers used for generation of *P. falciparum *expression vectors. Click here for file

Additional data file 2 The *P. falciparum *exportome. Click here for file

Additional data file 3 The exportomes of the *P. berghei *and *P. vivax *lineages. Click here for file

Additional data file 4 The ExportPred output from *C. hominis *and *T. parva*. Click here for file

## Figures and Tables

**Table 1:** Performance of ExportPred variants

Model number	1	2	3	4
PEXEL WMM	Default	Default	Hiller	Hiller
Signal sequence model	Default	SignalP	Default	SignalP
				
**Negative set**	**Positive set: **training sequences			
PfNegative	**0.98**	0.90	0.97	0.88
Simulated	**0.99**	0.95	0.99	0.95
Simulated (PfSS)	**0.96**	0.80	0.92	0.61
Simulated (SpSS)	**0.97**	0.50	0.95	0.16
Simulated (EPSS)	**0.95**	0.88	0.91	0.79
				
**Negative set**	**Positive set: **Rifins + Stevors			
PfNegative	0.96	**0.97**	0.95	0.94
Simulated	0.98	**0.99**	0.97	0.99
Simulated (PfSS)	**0.95**	0.93	0.91	0.77
Simulated (SpSS)	**0.95**	0.61	0.93	0.21
Simulated (EPSS)	0.91	**0.96**	0.89	0.91

Performance of ExportPred as measured by area under the respective ROC curve for combinations of model variant, and positive and negative dataset. For each pair of positive and negative sets, the best performing model is highlighted in bold. The four model variants are constructed by substituting ExportPred PEXEL weight model matrix (WMM) with the one published in [6,7] and/or by substituting the ExportPred signal sequence states with the HMM used in SignalP.

**Table 2:** *P. falciparum *gene families encoding exported proteins

Family	Paralogs	Transmembrane domains	Exons	Microarray data						Comments
					
				R	T	S	M	Sp	G	
PfEMP1	59	1	2	x	x	x	x	x	x	
DnaJ I	3	0	1/4/5	1	0	0	1	0	0	DnaJ domains 1-3
DnaJ III	16	0/1	2	3	1	1	3	0	0	DnaJ domain 1
EMP3	2	0	2	2	2	1	2	1	0	MC proteins
GBP130	3	0	2	2	2	2	1	1	0	RBC surface proteins
FIKK kinases	20	0	3	4	4	0	4	0	0	Protein kinase domain
PfMC-2TM	10	2	2	0	2	0	0	0	0	MC proteins
RIFIN	160	2	2	3	6	2	2	1	0	RBC surface proteins
STEVOR	30	2	2	0	0	0	0	0	0	MC proteins
a/b_HYDa	4	1 × 2, 2 × 1	1 × 1, 1 × 7, 2 × 2	0	0	0	0	0	0	a/b hydrolases
a/b_HYDb	4	0	2 × 1, 2 × 2	0	1	1	0	0	0	a/b hydrolases
HYP1	2	0	2	1	1	0	1	0	0	
HYP2	3	1 × 2, 2 × 0	1 × 1, 2 × 2	0	0	0	0	0	0	Probably not a real family
PHISTa	23	0	2/3	2	1	2	2	1	1	Four alpha helices
PHISTb	23	0	2	13	10	6	6	2	3	Four alpha helices
PHISTc	16	0	2	9	4	2	7	1	0	Four alpha helices conserved
HYP4	8	1	2	0	0	0	0	0	0	Similar to HYP6
HYP5	7	1	2	0	0	0	0	0	0	
HYP6	3	2	2	0	0	0	0	0	0	
HYP7	3	2	2	0	0	0	0	0	0	Similar to HYP8
HYP8	2	2	2	2	1	0	2	0	0	Proteomics iRBC localisation
HYP9	5	1	2	1	2	0	0	0	0	
HYP10	2	1	2	0	0	0	0	0	0	May be GPI anchored
HYP11	5	0	2 × 1, 3 × 2	0	1	0	0	0	0	Similar to PHIST
HYP12	3	0	2	2	1	1	1	0	0	
HYP13	2	2	2	1	0	0	1	0	0	Similar to HYP5
HYP15	4	2	2 × 1, 2 × 2	0	0	0	1	0	0	Similar to HYP5
HYP16	2	1 × 1, 1 × 2	2	1	0	0	1	0	0	Conserved
HYP17	2	1	2	1	0	0	0	0	0	

Approximately 75% of all 396 *P. falciparum *proteins predicted to be exported are organised in families. Counts in columns 5 to 10 represent the number of family members deemed to be expressed by this method for each life cycle stage (except PfEMP1). Abbreviations for life cycle stages in the microarray section are: R, ring; T, trophozoite; S, schizont; M, merozoite; Sp, sporozoite; G, gametocyte. GPI, glycosylphosphatidyl inositol; iRBC, infected red blood cell; MC, Maurer's clefts; RBC, red blood cell.

**Table 3:** A core complement of exported proteins is conserved across *Plasmodium*

Chromosome	Accession number	Exons	Transmembrane domain	Microarray data						Orthologs						Comments
					
				R	T	S	M	SP	G	Pv	Pk	Py	Pb	Pc	Pg	
1	PFA0210c	1	0	**7.42**	**8.70**	**10.16**	**10.65**	**7.88**	4.28	X		X		X	X	
	PFA0585w	1	0	2.99	3.07	3.06	2.99	3.06	3.05	X	X	X			X	
	PFA0610c	2 cl	0	2.65	2.71	2.70	2.65	2.70	2.69	X	X	X	X	X	X	Hyp11
2	PFB0105c	2 cl	0	**9.26**	4.41	**10.29**	**12.28**	**12.77**	2.31	X						PHISTc
	PFB0110w	2 cl	0	3.37	3.48	3.48	3.40	3.47	3.46	X						Hyp11
	PFB0900c	2 cl	0	**6.22**	3.21	3.23	**8.00**	3.21	3.20	X	X					PHISTc
	PFB0905c	2 cl	0	3.26	3.27	3.26	**6.34**	3.27	3.26	X	X					PHISTc
	PFB0910w	2 cl	2	2.73	2.80	2.79	3.72	2.80	2.78	X		X				
	PFB0915w	2 cl	1	**5.17**	3.43	3.97	**6.30**	3.42	3.40	X	X					LZ
3	PFC0075c	2 cl	0	3.13	3.21	3.20	3.15	3.20	3.19	X	X					1 SNP
	PFC0090w	2 cl	1	**6.51**	3.22	**6.31**	**10.36**	3.25	3.20	X	X	X	X	X		1 SNP
4	PFD0495c	2 cl	1	2.63	2.69	2.68	2.76	2.69	2.67	X	X	X	X	X	X	
5	PFE0055c	2 cl+2	0	2.37	2.37	2.35	**8.07**	2.37	2.36	X	X					DNAj typeI
	PFE1595c	2 cl	0	2.64	2.70	2.69	2.65	2.69	2.69	X						PHISTc
7	MAL7P1.172	2 cl	0	**9.65**	**6.52**	**5.03**	**9.47**	3.62	3.60	X						PHISTc
8	MAL8P1.4	2 cl	0	**5.46**	3.35	3.34	3.49	3.35	3.33	X						PHISTc
	PF08_0137	2 cl	0	**6.80**	**7.06**	4.16	**6.55**	4.14	4.13	X	X	X	X			PHISTc
9	PFI1725w	2 cl	0	3.84	3.36	3.35	**5.47**	3.36	3.33	X					X	
	PFI1750c	2 cl	0	2.71	7.53	2.68	2.64	2.68	2.67	X	X				X	Hyp11
	PFI1755c	2 cl	0	**12.20**	**9.89**	**9.11**	**11.33**	3.24	3.22	X						
	PFI1760w	2 cl	0	**9.44**	**7.41**	4.00	**9.63**	3.83	**7.03**	X						
	PFI1780w	2 cl	0	**8.72**	**6.92**	3.15	3.13	3.15	3.14	X	X					PHISTc
10	PF10_0021	2 cl	0	**6.01**	3.51	3.84	**5.38**	3.50	3.49	X						PHISTc
	PF10_0022	2 cl	0	3.00	3.02	3.00	3.18	3.02	3.00	X						PHISTc
	PF10_0023	2 cl	2	3.12	3.19	3.18	3.13	3.17	3.16	X	X					Hyp16
	PF10_0025	2 cl	1	**8.65**	2.84	3.64	**10.34**	2.86	2.85	X	X					Hyp16
	PF10_0161	2 cl	0	2.60	2.66	2.65	2.61	2.68	2.65	X						PHISTc
	PF10_0162	2 cl	0	3.41	3.50	3.50	3.78	3.49	3.48	X	X					PHISTc
	PF10_0163	2 cl	0	**5.26**	3.96	3.95	**7.43**	4.01	3.90	X	X					PHISTc
11	PF11_0503	2 cl	0	**7.08**	3.09	3.06	3.04	3.08	3.07	X	X					PHISTc
	PF11_0504	2 cl	0	3.50	3.55	3.55	3.51	3.55	3.54	X	X					Hyp11
12	PFL0045c	2 cl	0	3.14	3.21	3.20	3.18	3.20	3.18	X						PHISTc
	PFL0600w	1	0	3.07	3.16	3.22	3.08	3.22	3.14	X	X	X				
	PFL1660c	2 cl+2	0	2.49	2.55	2.53	2.51	2.55	2.53	X	X	X				
13	PF13_0076	2 cl	1	**6.85**	**6.35**	3.76	**6.28**	3.73	3.73	X						
14	PF14_0731	2 cl	0	**8.53**	**8.62**	3.42	3.34	3.53	3.41	X						PHISTc

For each conserved exported protein, this table presents exon structure, number of predicted transmembrane domains, protein localisation (where available), microarray expression (abbreviations same as in Table 2), conservation across the genus, and associated family. The text 'cl' in the exon column indicates a classic PEXEL structure with signal sequence in the first exon and PEXEL at the beginning of the second. For the microarray data group of columns, expression values for each member of the core list are presented. Values over 5 (indicating expression) in microarray data are highlighted in bold. Ortholog presence in other *Plasmodium *species is indicated by an X in the appropriate column of the Ortholog column group (Pv, *P. vivax*; Pk, *P. knowlesi*; Py, *P. yoelii*; Pb, *P. berghei*; Pc, *P. chabaudi*; Pg,*P. gallinaceum*). PlasmoDB IDs of genes conserved outside of the *P. vivax *and *P. falciparum *lineages are presented in bold.

## References

[B1] Waller KL, Cooke BM, Nunomura W, Mohandas N, Coppel RL (1999). Mapping the binding domains involved in the interaction between the *Plasmodium falciparum *knob-associated histidine-rich protein (KAHRP) and the cytoadherence ligand *P. falciparum* erythrocyte membrane protein 1 (PfEMP1).. J Biol Chem.

[B2] Freitas-Junior LH, Hernandez-Rivas R, Ralph SA, Montiel-Condado D, Ruvalcaba-Salazar OK, Rojas-Meza AP, Mancio-Silva L, Leal-Silvestre RJ, Gontijo AM, Shorte S, Scherf A (2005). Telomeric heterochromatin propagation and histone acetylation control mutually exclusive expression of antigenic variation genes in malaria parasites.. Cell.

[B3] Duraisingh MT, Voss TS, Marty AJ, Duffy MF, Good RT, Thompson JK, Freitas-Junior LH, Scherf A, Crabb BS, Cowman AF (2005). Heterochromatin silencing and locus repositioning linked to regulation of virulence genes in *Plasmodium falciparum*.. Cell.

[B4] Chen Q, Schlichtherle M, Wahlgren M (2000). Molecular aspects of severe malaria.. Clin Microbiol Rev.

[B5] Kyes SA, Rowe JA, Kriek N, Newbold CI (1999). Rifins: a second family of clonally variant proteins expressed on the surface of red cells infected with *Plasmodium falciparum*.. Proc Natl Acad Sci USA.

[B6] Marti M, Good RT, Rug M, Knuepfer E, Cowman AF (2004). Targeting malaria virulence and remodeling proteins to the host erythrocyte.. Science.

[B7] Hiller NL, Bhattacharjee S, van Ooij C, Liolios K, Harrison T, Lopez-Estrano C, Haldar K (2004). A host-targeting signal in virulence proteins reveals a secretome in malarial infection.. Science.

[B8] Bendtsen JD, Nielsen H, von Heijne G, Brunak S (2004). Improved prediction of signal peptides: SignalP 3.0.. J Mol Biol.

[B9] Rabiner LR (1989). A tutorial on hidden Markov models and selected applications inspeech recognition.. Proc IEEE.

[B10] Knuepfer E, Rug M, Cowman AF (2005). Function of the plasmodium export element can be blocked by green fluorescent protein.. Mol Biochem Parasitol.

[B11] Withers-Martinez C, Jean L, Blackman MJ (2004). Subtilisin-like proteases of the malaria parasite.. Mol Microbiol.

[B12] Bender A, van Dooren GG, Ralph SA, McFadden GI, Schneider G (2003). Properties and prediction of mitochondrial transit peptides from *Plasmodium falciparum*.. Mol Biochem Parasitol.

[B13] Bozdech Z, Llinas M, Pulliam BL, Wong ED, Zhu J, DeRisi JL (2003). The transcriptome of the intraerythrocytic developmental cycle of *Plasmodium falciparum*.. PLoS Biol.

[B14] Le Roch KG, Zhou Y, Blair PL, Grainger M, Moch JK, Haynes JD, De La Vega P, Holder AA, Batalov S, Carucci DJ, Winzeler EA (2003). Discovery of gene function by expression profiling of the malaria parasite life cycle.. Science.

[B15] Fernandez V, Hommel M, Chen Q, Hagblom P, Wahlgren M (1999). Small, clonally variant antigens expressed on the surface of the *Plasmodium falciparum*-infected erythrocyte are encoded by the rif gene family and are the target of human immune responses.. J Exp Med.

[B16] Cheng Q, Cloonan N, Fischer K, Thompson J, Waine G, Lanzer M, Saul A (1998). stevor and rif are *Plasmodium falciparum *multicopy gene families which potentially encode variant antigens.. Mol Biochem Parasitol.

[B17] Sam-Yellowe TY, Florens L, Johnson JR, Wang T, Drazba JA, Le Roch KG, Zhou Y, Batalov S, Carucci DJ, Winzeler EA, Yates JR (2004). A Plasmodium gene family encoding Maurer's cleft membrane proteins: structural properties and expression profiling.. Genome Res.

[B18] Schneider AG, Mercereau-Puijalon O (2005). A new Apicomplexa-specific protein kinase family: multiple members in *Plasmodium falciparum*, all with an export signature.. BMC Genomics.

[B19] Ward P, Equinet L, Packer J, Doerig C (2004). Protein kinases of the human malaria parasite *Plasmodium falciparum: *the kinome of a divergent eukaryote.. BMC Genomics.

[B20] Xu P, Widmer G, Wang Y, Ozaki LS, Alves JM, Serrano MG, Puiu D, Manque P, Akiyoshi D, Mackey AJ (2004). The genome of *Cryptosporidium hominis*.. Nature.

[B21] Guerin-Marchand C, Druilhe P, Galey B, Londono A, Patarapotikul J, Beaudoin RL, Dubeaux C, Tartar A, Mercereau-Puijalon O, Langsley G (1987). A liver-stage-specific antigen of *Plasmodium falciparum *characterized by gene cloning.. Nature.

[B22] Biggs BA, Kemp DJ, Brown GV (1989). Subtelomeric chromosome deletions in field isolates of *Plasmodium falciparum *and their relationship to loss of cytoadherence *in vitro*.. Proc Natl Acad Sci USA.

[B23] Lanzer M, Wertheimer SP, de Bruin D, Ravetch JV (1994). Chromatin structure determines the sites of chromosome breakages in *Plasmodium falciparum*.. Nucleic Acids Res.

[B24] Pologe LG, Ravetch JV (1988). Large deletions result from breakage and healing of *P. falciparum *chromosomes.. Cell.

[B25] Escalante AA, Barrio E, Ayala FJ (1995). Evolutionary origin of human and primate malarias: evidence from the circumsporozoite protein gene.. Mol Biol Evol.

[B26] Fischer K, Chavchich M, Huestis R, Wilson DW, Kemp DJ, Saul A (2003). Ten families of variant genes encoded in subtelomeric regions of multiple chromosomes of *Plasmodium chabaudi*, a malaria species that undergoes antigenic variation in the laboratory mouse.. Mol Microbiol.

[B27] del Portillo HA, Fernandez-Becerra C, Bowman S, Oliver K, Preuss M, Sanchez CP, Schneider NK, Villalobos JM, Rajandream MA, Harris D (2001). A superfamily of variant genes encoded in the subtelomeric region of *Plasmodium vivax*.. Nature.

[B28] The Wellcome Trust Sanger Institute Plasmodium Sequence Data. ftp://ftp.sanger.ac.uk/pub/pathogens/Plasmodium.

[B29] Shi J, Blundell TL, Mizuguchi K (2001). FUGUE: sequence-structure homology recognition using environment-specific substitution tables and structure-dependent gap penalties.. J Mol Biol.

[B30] Favaloro JM, Coppel RL, Corcoran LM, Foote SJ, Brown GV, Anders RF, Kemp DJ (1986). Structure of the RESA gene of *Plasmodium falciparum*.. Nucleic Acids Res.

[B31] Bork P, Sander C, Valencia A, Bukau B (1992). A module of the DnaJ heat shock proteins found in malaria parasites.. Trends Biochem Sci.

[B32] Watanabe J (1997). Cloning and characterization of heat shock protein DnaJ homologues from *Plasmodium falciparum *and comparison with ring infected erythrocyte surface antigen.. Mol Biochem Parasitol.

[B33] Walsh P, Bursac D, Law YC, Cyr D, Lithgow T (2004). The J-protein family: modulating protein assembly, disassembly and translocation.. EMBO Rep.

[B34] The Plasmodium Genome Resource. http://www.plasmodb.org.

[B35] Eddy SR (1998). Profile hidden Markov models.. Bioinformatics.

[B36] Kall L, Krogh A, Sonnhammer EL (2004). A combined transmembrane topology and signal peptide prediction method.. J Mol Biol.

[B37] Sonnhammer EL, von Heijne G, Krogh A (1998). A hidden Markov model for predicting transmembrane helices in protein sequences.. Proc Int Conf Intell Syst Mol Biol.

[B38] Foth BJ, Ralph SA, Tonkin CJ, Struck NS, Fraunholz M, Roos DS, Cowman AF, McFadden GI (2003). Dissecting apicoplast targeting in the malaria parasite *Plasmodium falciparum*.. Science.

[B39] Long M, de Souza SJ, Rosenberg C, Gilbert W (1996). Exon shuffling and the origin of the mitochondrial targeting function in plant cytochrome c1 precursor.. Proc Natl Acad Sci USA.

[B40] Kilian O, Kroth PG (2005). Identification and characterization of a new conserved motif within the presequence of proteins targeted into complex diatom plastids.. Plant J.

[B41] Tordai H, Patthy L (2004). Insertion of spliceosomal introns in proto-splice sites: the case of secretory signal peptides.. FEBS Lett.

[B42] Rehmany AP, Gordon A, Rose LE, Allen RL, Armstrong MR, Whisson SC, Kamoun S, Tyler BM, Birch PR, Beynon JL (2005). Differential recognition of highly divergent downy mildew avirulence gene alleles by RPP1 resistance genes from two *Arabidopsis* lines.. Plant Cell.

[B43] Wickham ME, Rug M, Ralph SA, Klonis N, McFadden GI, Tilley L, Cowman AF (2001). Trafficking and assembly of the cytoadherence complex in *Plasmodium falciparum*-infected human erythrocytes.. EMBO J.

[B44] The National Center for Biotechnology Information. http://www.ncbi.nlm.nih.gov.

[B45] Fujioka H, Millet P, Maeno Y, Nakazawa S, Ito Y, Howard RJ, Collins WE, Aikawa M (1994). A nonhuman primate model for human cerebral malaria: rhesus monkeys experimentally infected with Plasmodium fragile.. Exp Parasitol.

[B46] Horrocks P, Pinches RA, Chakravorty SJ, Papakrivos J, Christodoulou Z, Kyes SA, Urban BC, Ferguson DJ, Newbold CI (2005). PfEMP1 expression is reduced on the surface of knobless *Plasmodium falciparum *infected erythrocytes.. J Cell Sci.

[B47] Kumar N, Koski G, Harada M, Aikawa M, Zheng H (1991). Induction and localization of *Plasmodium falciparum *stress proteins related to the heat shock protein 70 family.. Mol Biochem Parasitol.

[B48] Banumathy G, Singh V, Tatu U (2002). Host chaperones are recruited in membrane-bound complexes by *Plasmodium falciparum*.. J Biol Chem.

[B49] Rug M, Wickham ME, Foley M, Cowman AF, Tilley L (2004). Correct promoter control is needed for trafficking of the ring-infected erythrocyte surface antigen to the host cytosol in transfected malaria parasites.. Infect Immun.

[B50] Blisnick T, Morales Betoulle ME, Barale JC, Uzureau P, Berry L, Desroses S, Fujioka H, Mattei D, Braun Breton C (2000). Pfsbp1, a Maurer's cleft *Plasmodium falciparum *protein, is associated with the erythrocyte skeleton.. Mol Biochem Parasitol.

[B51] LaCount DJ, Vignali M, Chettier R, Phansalkar A, Bell R, Hesselberth JR, Schoenfeld LW, Ota I, Sahasrabudhe S, Kurschner C (2005). A protein interaction network of the malaria parasite *Plasmodium falciparum*.. Nature.

[B52] Young JA, Fivelman QL, Blair PL, de la Vega P, Le Roch KG, Zhou Y, Carucci DJ, Baker DA, Winzeler EA (2005). The *Plasmodium falciparum *sexual development transcriptome: a microarray analysis using ontology-based pattern identification.. Mol Biochem Parasitol.

[B53] Silvestrini F, Bozdech Z, Lanfrancotti A, Di Giulio E, Bultrini E, Picci L, Derisi JL, Pizzi E, Alano P (2005). Genome-wide identification of genes upregulated at the onset of gametocytogenesis in *Plasmodium falciparum*.. Mol Biochem Parasitol.

[B54] Eksi S, Haile Y, Furuya T, Ma L, Su X, Williamson KC (2005). Identification of a subtelomeric gene family expressed during the asexual-sexual stage transition in *Plasmodium falciparum*.. Mol Biochem Parasitol.

[B55] Florens L, Liu X, Wang Y, Yang S, Schwartz O, Peglar M, Carucci DJ, Yates JR, Wub Y (2004). Proteomics approach reveals novel proteins on the surface of malaria-infected erythrocytes.. Mol Biochem Parasitol.

[B56] Daily JP, Le Roch KG, Sarr O, Ndiaye D, Lukens A, Zhou Y, Ndir O, Mboup S, Sultan A, Winzeler EA, Wirth DF (2005). *In vivo* transcriptome of *Plasmodium falciparum *reveals overexpression of transcripts that encode surface proteins.. J Infect Dis.

[B57] Florens L, Washburn MP, Raine JD, Anthony RM, Grainger M, Haynes JD, Moch JK, Muster N, Sacci JB, Tabb DL (2002). A proteomic view of the *Plasmodium falciparum *life cycle.. Nature.

[B58] Lasonder E, Ishihama Y, Andersen JS, Vermunt AM, Pain A, Sauerwein RW, Eling WM, Hall N, Waters AP, Stunnenberg HG (2002). Analysis of the *Plasmodium falciparum *proteome by high-accuracy mass spectrometry.. Nature.

[B59] Sanders PR, Gilson PR, Cantin GT, Greenbaum DC, Nebl T, Carucci DJ, McConville MJ, Schofield L, Hodder AN, Yates JR (2005). Distinct protein classes including novel merozoite surface antigens in raft-like membranes of *Plasmodium falciparum*.. J Biol Chem.

[B60] Gardner MJ, Hall N, Fung E, White O, Berriman M, Hyman RW, Carlton JM, Pain A, Nelson KE, Bowman S (2002). Genome sequence of the human malaria parasite *Plasmodium falciparum*.. Nature.

[B61] Carlton JM, Angiuoli SV, Suh BB, Kooij TW, Pertea M, Silva JC, Ermolaeva MD, Allen JE, Selengut JD, Koo HL (2002). Genome sequence and comparative analysis of the model rodent malaria parasite *Plasmodium yoelii yoelii*.. Nature.

[B62] Carlton JM, Vinkenoog R, Waters AP, Walliker D (1998). Gene synteny in species of *Plasmodium*.. Mol Biochem Parasitol.

[B63] Carlton JM, Galinski MR, Barnwell JW, Dame JB (1999). Karyotype and synteny among the chromosomes of all four species of human malaria parasite.. Mol Biochem Parasitol.

[B64] Freitas-Junior LH, Bottius E, Pirrit LA, Deitsch KW, Scheidig C, Guinet F, Nehrbass U, Wellems TE, Scherf A (2000). Frequent ectopic recombination of virulence factor genes in telomeric chromosome clusters of *P. falciparum*.. Nature.

[B65] Volkman SK, Hartl DL, Wirth DF, Nielsen KM, Choi M, Batalov S, Zhou Y, Plouffe D, Le Roch KG, Abagyan R (2002). Excess polymorphisms in genes for membrane proteins in *Plasmodium falciparum*.. Science.

[B66] Janssen CS, Phillips RS, Turner CM, Barrett MP (2004). *Plasmodium* interspersed repeats: the major multigene superfamily of malaria parasites.. Nucleic Acids Res.

[B67] GeneDB: Genome Database of the Wellcome Trust Sanger Institute Pathogen Sequencing Unit. http://www.geneDB.org.

[B68] Bowman AW, Azzalini A (1997). Applied Smoothing Techniques for Data Analysis.

[B69] Swets JA (1988). Measuring the accuracy of diagnostic systems.. Science.

[B70] Benjamini Y, Drai D, Elmer G, Kafkafi N, Golani I (2001). Controlling the false discovery rate in behavior genetics research.. Behav Brain Res.

[B71] Hirsh AE, Fraser HB (2001). Protein dispensability and rate of evolution.. Nature.

[B72] Jordan IK, Rogozin IB, Wolf YI, Koonin EV (2002). Essential genes are more evolutionarily conserved than are nonessential genes in bacteria.. Genome Res.

[B73] Edgar RC (2004). MUSCLE: multiple sequence alignment with high accuracy and high throughput.. Nucleic Acids Res.

[B74] Irizarry RA, Hobbs B, Collin F, Beazer-Barclay YD, Antonellis KJ, Scherf U, Speed TP (2003). Exploration, normalization, and summaries of high density oligonucleotide array probe level data.. Biostatistics.

[B75] Wu Z, Irizarry RA (2005). Stochastic models inspired by hybridization theory for short oligonucleotide arrays.. J Comput Biol.

[B76] Bioconductor: Software for the Analysis and Comprehension of Genomic Data. http://www.bioconductor.org.

[B77] Schmidt HA, Strimmer K, Vingron M, von Haeseler A (2002). TREE-PUZZLE: maximum likelihood phylogenetic analysis using quartets and parallel computing.. Bioinformatics.

[B78] Jones DT (1999). Protein secondary structure prediction based on position-specific scoring matrices.. J Mol Biol.

[B79] van Dooren GG, Marti M, Tonkin CJ, Stimmler LM, Cowman AF, McFadden GI (2005). Development of the endoplasmic reticulum, mitochondrion and apicoplast during the asexual life cycle of *Plasmodium falciparum*.. Mol Microbiol.

[B80] Wu Y, Kirkman LA, Wellems TE (1996). Transformation of *Plasmodium falciparum *malaria parasites by homologous integration of plasmids that confer resistance to pyrimethamine.. Proc Natl Acad Sci USA.

[B81] Trager W, Jensen JB (1976). Human malaria parasites in continuous culture.. Science.

[B82] The Institute for Genomic Research. http://www.tigr.org.

[B83] The Toxoplasma Genome Resource. http://www.toxodb.org.

[B84] Cuff JA, Barton GJ (2000). Application of multiple sequence alignment profiles to improve protein secondary structure prediction.. Proteins.

[B85] Jpred: a Consensus Method for Protein Secondary Structure Prediction. http://www.compbio.dundee.ac.uk/~www-jpred.

