# Supplementary material for: Lineage-specific expansion of proteins exported to erythrocytes in malaria parasites
Source: Genome Biol. 2006 Feb 20;7(2):R12. doi: 10.1186/gb-2006-7-2-r12 (PMC1431722; doi:10.1186/gb-2006-7-2-r12)
Supplement: Additional data file 1 — Primers used for generation of P. falciparum expression vectors. [file gb-2006-7-2-r12-S1.doc]

| **Primer** | Sequence |
| --- | --- |
| PFI1755c-s | CACCataaaaaaATGCAAACCCGTAAAT |
| PFI1755c-as | TGTTTTTTTTAAATCCTGTTCTT |
| PFE0055c-s | CACCgttcaataATGTCCATTTTAAATAAATACGAA |
| PFE0055c-as | cttagaactttctcttgatgat |
| PFI1780w-s | CACCgaaaagaaATGGCTGTTAGTACA |
| PFI1780w-as | acttcttaaattgttatgttcttc |
| PFE0360c-s | CACCataaaaaagATGATATACTT |
| PFE0360c-as | tccttttgatgataaatcattatt |
| PF10_0321-s | CACCttaaggggATGCTTAAACATGTATTT |
| PF10_0321-as | TATTACATCATCAACCTTTT |
| PF14_0607-s | CACCtatagaatATGCTGATGTTGTAT |
| PF14_0607-as | caacttatctaagtttgagaa |
| PFE0355c-s | CACCaatatagaATGATAAATAGACAGT |
| PFE0355c-as | tcctttaatactattatttttataa |
